# Interventions for the management of concomitant COPD and hypertension: A systematic review

**DOI:** 10.1177/26335565251341389

**Published:** 2025-06-23

**Authors:** Sadan Taher, Aletta E. Schutte, John R. Hurst, Chris P. Gale, Sameera Ansari

**Affiliations:** 1 School of Clinical Medicine, Faculty of Medicine and Health, 7800 UNSW Sydney, Sydney, NSW, Australia; 2 School of Population Health, Faculty of Medicine and Health, 7800 UNSW Sydney, Sydney, NSW, Australia; 3 George Institute for Global Health, Sydney, NSW, Australia; 4 UCL Respiratory, 4919 University College London, London, UK; 5 Leeds Institute of Cardiovascular and Metabolic Medicine, 4468 University of Leeds, Leeds, UK; 6 Leeds Institute for Data Analytics, 4468 University of Leeds, Leeds, UK; 7 Department of Cardiology, Leeds Teaching Hospitals NHS Trust, Leeds, UK; 8 Woolcock Institute of Medical Research, Macquarie University, Sydney, NSW, Australia; 9 Faculty of Health Sciences and Medicine, Bond University, Gold Coast, QLD, Australia

**Keywords:** COPD, hypertension, multimorbidity, systematic review

## Abstract

**Background:** Chronic obstructive pulmonary disease (COPD) and hypertension are prevalent public health burdens, with hypertension often co-existing in up to 65% of COPD patients and complicating patient management. While numerous clinical guidelines address these conditions individually, there is a scarcity of evidence-based interventions for managing both simultaneously.

**Purpose:** This systematic review aimed to identify interventional studies targeting people with concomitant COPD and hypertension

**Research Design:** The review followed PRISMA guidelines and was registered on PROSPERO (CRD42024533767). A comprehensive search was conducted across multiple databases, including PubMed, EMBASE, Scopus, CINAHL, the Cochrane Library and Cochrane Controlled Register of Trials.

**Results:** The search yielded 3,348 records, of which three studies met the inclusion criteria. These studies examined interventions including oral nitrate supplementation, medication adherence management and a collaborative care model. One study reported a significant reduction in systolic blood pressure (SBP) and improvement in COPD-related outcomes, while the other two reported mixed effects. The collaborative care model notably reduced hospitalizations and healthcare costs.

**Conclusions:** The findings highlight the limited and inconsistent evidence available for managing concomitant COPD and hypertension, reinforcing the need for further research on this topic. Despite frequent clinical encounters with patients having both conditions, practitioners lack a unified treatment strategy. Future studies should focus on developing comprehensive management approaches that address the complex interplay between COPD and hypertension, aiming to improve patients’ health outcomes and deliver efficient healthcare.

## Introduction

COPD and hypertension are both major public health burdens globally, with an economic burden of 74.4 million and 393 million disability-adjusted life-years, respectively, in 2019.^1^ Hypertension and COPD commonly co-exist, hypertension being the most prevalent co-morbidity of COPD occurring in up to 65% of cases.^2,3^ Patients with COPD are at an increased risk of cardiovascular disease, especially as they exhibit chronic systemic inflammation, a factor also common to the pathophysiology of hypertension.^4,5^ COPD also exacerbates hypertension-mediated organ damage and cardiorenal impairment- both of which need to be considered when treating COPD patients who have co-existing hypertension.^4,6,7^ Therefore, evidence on accurately managing concomitant COPD and hypertension is needed.

The 2024 GOLD COPD report advises that hypertension should be treated according to usual guidelines despite the co-existence with COPD, given lack of evidence of selective beta-blockers increasing cardiovascular risk or reducing the efficacy of long-acting beta agonists (LABAs)^8–10^ in this population. The 2023 European Society of Hypertension acknowledges the significant burden of concomitant COPD and hypertension but recommends general guidelines for hypertension without a specific regimen for co-existing COPD.^11^ This is an update from the previous guidelines which contra-indicated beta-blockers for COPD patients. Despite being recommended in the Australian and New Zealand COPD-X Plan, beta blockers remain under-prescribed in managing patients with concomitant COPD and hypertension.^12–15^ In an Australian retrospective cohort study, only 45% patients with known cardiac disease received beta blocker therapy, proving practitioners are not confident in managing patients with concomitant COPD and hypertension.^14^ The 2017 American Heart Association (AHA) guidelines do not include a management approach in the co-existence of COPD except in one situation.^16^ Although it is known that non-selective beta blockers can induce bronchoconstriction in patients with COPD, the AHA guidelines do not provide an alternative approach to concomitant COPD and hypertension, despite being a common presentation.^16,17^

Given how COPD and hypertension commonly co-exist, it is important that clinical practitioners encountering patients with both conditions are provided with more specific guidance for management. An extensive background search of literature yielded only two studies that investigated people with both COPD and hypertension, highlighting the need for specific interventions to simultaneously manage both conditions. Therefore, the aim of this systematic review was conducted to identify interventions targeted at managing people with concomitant COPD and hypertension.

## Methods

This systematic review was conducted according to the PRISMA (Preferred Reporting Items for Systematic Reviews and Meta-analyses) guidelines.^18^ The review was prospectively registered on the PROSPERO register of systematic reviews (Registration No. CRD42024533767).

### Search strategy

**Figure 1.**
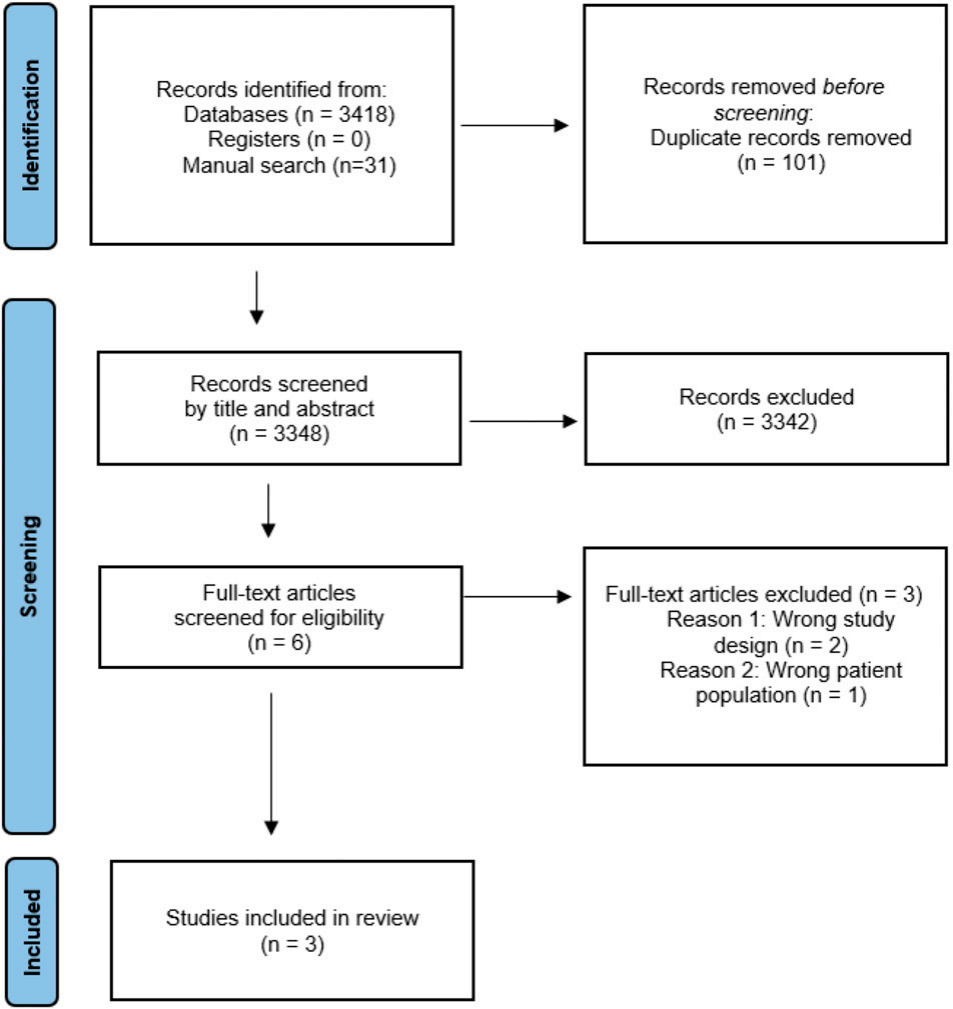
PRISMA flow of search strategy used in the review.

The following databases were searched for interventional studies, limited to English

and with no date limit: PubMed, EMBASE, CINAHL, Scopus, the Cochrane library, and Cochrane Controlled Register of Trials. The search strategy for all databases has been included in Supplement 1; the final search was conducted on 17^th^ April 2024. Trial registries including the International Standard Randomised Controlled Trial Number (ISRCTN), and the Australian New Zealand Clinical Trials Registry (ANZCTR), which also includes ClinicalTrials.gov, were searched manually for any relevant results. An additional manual search was also performed by checking the reference lists of eligible papers and including any relevant references. The process of study selection and the results yielded at each stage of the systematic review are presented in Figure 1. An expert librarian at UNSW Sydney was consulted by ST for guidance on developing and refining the search strategy.

### Selection criteria

To be included in the review, studies had to recruit adults aged 18 years and above. The interventions implemented in the studies had to target the management of people with a diagnosis of co- existing COPD AND hypertension. Studies that included interventions for managing people with either COPD OR hypertension were not included. Comparator arms were no intervention or usual care of COPD and/or hypertension. The main outcome measures of interest were changes in SBP and/or DBP (diastolic blood pressure). Other outcomes of interest included change in 6-min walk distance (6MWD), Asthma Control Questionnaire (ACQ) scores, and Clinical COPD Questionnaire (CCQ) scores.

### Data collection and extraction

The types of studies considered were interventional studies for concomitant COPD and hypertension, such as randomised controlled trials, single group pre and post-test, feasibility and pilot studies.

All results from the databases were imported into EndNote and duplicates were removed, following which the records were uploaded to the Covidence online software. Registry search was then completed, but no results were yielded to import into Covidence. Titles and abstracts of all uploaded search records were then independently screened by two researchers (ST and SA), blinded to each other’s decisions. Any conflicts were resolved by a third member of the team (AES).

A data extraction template was created, incorporating various parameters of the systematic review using the PICO (Population, Intervention, Control, Outcome) format, details of which were included in the above-mentioned eligibility criteria. After screening the titles and abstracts, eligible full-text papers were screened. Each full text was reviewed by two researchers (ST and AES), and any reason for exclusion was recorded (shown in PRISMA flowchart). Any conflicts were resolved by a third member of the team (SA). The final selection of studies included in the review consisted of those meeting the inclusion criteria and having adequate data for extraction. Data was extracted from the final eligible studies by ST.

### Risk of bias assessment

The quality of the eligible studies was assessed in accordance with the Cochrane Handbook for Systematic Review of Interventions, using the Cochrane Risk of bias tool.^19^ Studies were considered as having a high risk of bias if one or more domains were calculated as high risk.

## Results

A total of 3,348 records were included for screening by title and abstracts after removing duplicates. Six records were included for full-text review and of those, three were included for data extraction, outlined previously in Figure 1. Reasons for exclusion included the wrong study design and the wrong patient population. Two studies included were randomised controlled trials, while one study included a retrospective analysis for the placebo group. Study duration ranged from twelve weeks up to one year.^20,21^

### Characteristics of the included studies

Study characteristics and demographics are summarised in Table 1. Only three studies investigated patients who had both COPD and hypertension.^20–22^ The studies included a control group of participants who did not receive the intervention or received usual care, that is, typical care for COPD and hypertension managed separately.

**Table 1.** Summary of characteristics of included research studies.

Study	Country	Setting	Design	Participants	Duration (months)	RoB
1. Alasmari et al., 2024	United Kingdom	Participants’ home	RCT	Adults (18+) who have had stable COPD without an acute exacerbation or change in medication in the preceding month and have a baseline SBP ⩾130 mmHg	3	Low
2. Torres-Robles et al., 2021	Spain	Community pharmacies	Cluster RCT	Adult patients (18+) suffering from hypertension, asthma and COPD were recruited	6	Some concerns
3. Matzke et al., 2018	USA	Hospital wards/ Community pharmacies	Retrospective data analysis (for control group)	Adult patients (18+) with a documented diagnosis of 2 or more of the 7 targeted chronic conditions (congestive heart failure, hypertension, hyperlipidemia, diabetes mellitus, asthma, chronic obstructive pulmonary disease, and depression), prescriptions for 4 or more medications, and having a primary care physician in the Carilion Clinic health system	12	High

Study 1 by Alasmari et al., was a randomised, double-blind, placebo-controlled parallel group trial conducted in the UK, which assessed the effect of oral nitrate supplementation in beetroot juice in lowering blood pressure, enhancing endothelial function and improving exercise capacity in people with COPD.^20^

Study 2 by Torres-Robles et al., was a cluster randomised controlled trial assessing the effectiveness of a medication adherence management intervention on blood pressure and COPD symptom intensity in patients from 98 pharmacies in Spain.^22^ The intervention was based on theoretical frameworks proven to change patient behaviour.

Study 3 by Matzke et al., investigated the impact of a pharmacist-physician collaborative care model on patient outcomes, including blood pressure levels, and health services utilisation in the USA.^21^

### Intervention characteristics

The interventions were heterogenous, with varying durations and health settings. A summary of the intervention, comparator and outcome is presented in Table 2.

**Table 2.** Summary of results of included studies.

Study	Intervention	Comparator	Outcomes		
			BP	COPD	Healthcare/costs
1. Alasmari et al., 2024	Dietary oral nitrate supplementation in the form of beetroot juice	Placebo, normal beetroot juice	A significant decrease in systolic blood pressure compared with placebo (−4.5mmHg, 95% CI [−3.0−5.9]; p<0.001) No significant decrease in mean arterial pressure (- 1.67mmHg, 95% CI [-1.81- 0.04]; p=0.07), nor diastolic blood pressure (−1.1mmHg, 95% CI [−3.1–1.0]; p=0.31)	A significant improvement in 6MWD in participants receiving active treatment (+30.0m, 95% CI [15.7-44.2]; p<0.0001) No significant difference in the CAT score (−1, 95% CI [- 3-0]; p=0.09)	-
2. Torres-Robles et al., 2021	Medication adherence management intervention in community pharmacies	Usual care	Increase in the percentage of adherent patients, reaching 90% in the intervention group by the end of the study No significant changes in systolic blood pressure (−1.10mmHg, 95% CI [-4.49 - 2.29]; p=0.53)	Significant reduction in the mean CCQ scores (0.58 points)	-
3. Matzke et al., 2018	Pharmacist-physician collaborative care model	Usual care	Significant decrease in systolic blood pressure (- 6.28mmHg, 95% CI [-0.2-2.30]; p<0.0001)	-	31.2% decrease in hospitalisations during the 12-month post-intervention Cost reduction of $5,156,675 in the collaborative care group compared to only $475,071 for hospitalisations in the usual care group

In Study 1, participants were allocated to placebo or active treatment groups via an online randomisation system.^20^ Both the placebo and intervention group received daily beetroot juice for the 12-week duration of the study. They were also asked to not alter their usual diet or lifestyle, and to comply with their prescribed antihypertensives during the study period.

For Study 2, patients were recruited from pharmacies across six Spanish provinces.^22^ Participants, blinded to group allocation, attended monthly face-to-face visits for six months. The intervention group received a pharmacist-delivered, protocol-based medication adherence management intervention based on behaviour change frameworks.^23,24^ After identifying barriers to medication adherence,^25,26^ strategies were proposed to target them and progress reviewed in follow-up visits.^27^

Participants for Study 3 were recruited via an electronic medical record-based algorithm and/or referral from an independent physician or care coordinator.^21^ Participants received a comprehensive,^28^ tailored medication and chronic disease management as part of a collaborative care model by a multidisciplinary team.^29,30^ A 15-30 minute office visit was scheduled with a clinical pharmacist within 72 hours of discharge for participants in the collaborative care group with medication-related issues.

### Outcome measures

The primary outcome measure of Study 1 was change in SBP, measured by the participants using a home monitor over the first 4 days of the experiment and again at the end of the 12 weeks.^20^ Secondary outcomes included change in 6MWD and measures of endothelial function, including reactive hyperaemia index and augmentation index, as well as platelet function and plasma nitrate.^20^

In Study 2, medication adherence was the primary outcome assessed using MGL-MAQ and expressed as a percentage of adherent participants.^22^ Secondary outcomes included COPD control, assessed using the CCQ. Hypertension control was also measured and reported as the mean scores of SBP and DBP.^22^

In Study 3, the primary outcomes were change in diabetes, hypertension, and hyperlipidaemia.^21^ In the interest of this systematic review, changes in hypertension were assessed through differences in SBP and DBP. Secondary outcomes were the same measures but assessed for participants whose baseline was above goal (i.e. uncontrolled disease).^21^ Changes in the number of emergency department visits, hospitalisations, and the costs associated with them were also measured.^21^

### Effectiveness of study interventions

In Study 1 by Alasmari et al., oral nitrate supplementation was associated with a significant decrease in SBP compared with placebo.^20^ There was no significant decrease between groups in mean arterial pressure or COPD Assessment Test (CAT) scores, but there was a significant improvement in 6MWD in the treatment group.

Study 2 found an overall significant increase in medication adherence by 51.8% in the intervention group when compared to 22.2% in the control group.^22^ By the end of six months’ follow-ups, 90% of patients were adherent to their prescribed medication, doubling the baseline percentage in the intervention group. Adherence in the control group remained relatively constant during the following visits, always below 70%. Although there was no significant change in SBP between groups, there was a statistically significant reduction of 0.58 points in the mean CCQ scores.

Study 3 measured similar clinical outcomes,^21^ and led to significant decrease in SBP amongst the collaborative care group compared to participants receiving usual care. The study also found a 31.2% decrease in hospitalisations at 12 months’ follow-up in the intervention group, significantly greater than the 8.7% decrease in the usual care group.

**Figure 2.**
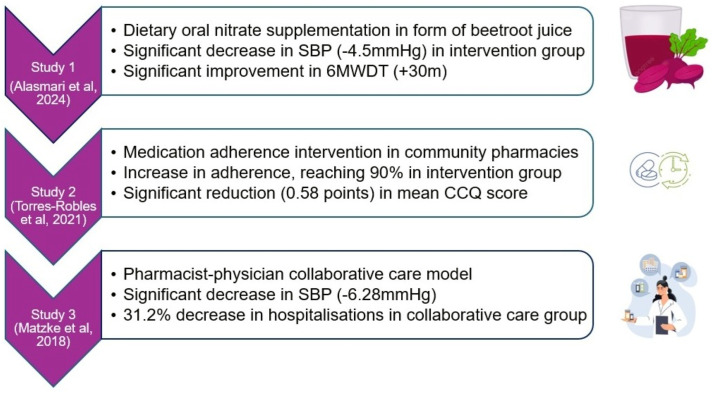
Summary of main findings of the systematic review.

Given the limited number of studies and their significant heterogeneity, a meta-analysis could not be conducted to assess the effectiveness of the interventions in improving outcome measures.^31^ The main findings of the systematic review have been summarized in Figure 2.

### Risk of bias

Using the Cochrane risk of bias tool, Study 1 was classified as low risk of bias, Study 2 had some concerns and Study 3 had a high risk of bias.^19^ Further details are outlined in Supplement 2.

## Discussion

This review found few studies examining interventions for co-existent hypertension and COPD, and conflicting results regarding effectiveness of the interventions that had been tested. Two studies established statistically significant reductions in SBP in the intervention group while one failed to do so. Additionally, two interventions reported COPD clinical benefits including an improvement in 6MWD distance, medication adherence, and decrease in hospitalisations.

The main finding of Alasmari’s trial (Study 1) is that a 12-week dietary nitrate supplementation, in the form of beetroot juice, leads to a sustained decrease in SBP and improves exercise capacity in people with COPD and hypertension.^20^ The study also showed improvement in measures of cardiovascular risk including endothelial function and arterial stiffness. Although previous studies have suggested potential beneficial effects of beetroot juice as a form of dietary nitrate supplementation in hypertensive patients, none looked at patients with both COPD and hypertension.^32-34^ A prior study, for example, improved central but not peripheral SBP in participants with type 2 diabetes.^35^ Another randomised controlled trial included only patients with COPD (the ON-EPIC study) and demonstrated a decrease in SBP by 5±3.7 mmHg with 8-wwek dietary nitrate supplementation.^36^ Therefore, a study targeting a patient population of concomitant COPD and hypertension, was needed to investigate the effectiveness of dietary nitrate supplementation. Study 1 offers this and has proven its strength with effectiveness of the intervention, a relatively low dropout rate and high adherence to the intervention.^20^

However, there remains evidence contradicting the findings of Study 1, with a 5-week study in Sweden not showing any reduction in SBP in 50–70-year-old hypertensive participants.^37^ Although this may have been partly due to a low dose of nitrate, the improvement in SBP seen in this study remains insufficient to be used as a primary method of treating people with concomitant COPD and hypertension. The trial also recruited a small sample size of 81 participants, mostly of Caucasian ethnicity, who were asked to manually measure their BP using a home monitor which may mitigate external factors like ‘the white coat phenomenon’, but also poses a risk of inaccuracy as it is unclear whether participants were educated on taking BP readings. Additionally, the study excluded patients that received more than three anti-hypertensives, although it is a common combination many hypertensive patients receive. Having demonstrated overall efficacy in Study 1, longer-term, multi-centre studies are needed to examine the effects of oral nitrate on cardiovascular and pulmonary disease events.

Study 2 established that a community pharmacist medication adherence intervention improves medication adherence and clinical outcomes.^22^ Although there has been previous literature that presented the effectiveness of pharmacist-led interventions on co-existing chronic conditions, none targeted concomitant COPD and hypertension.^38,39^ In Study 2, the observed baseline percentage of adherent patients, close to 50%, was consistent with the figures previously reported by the WHO to describe adherence to long-term therapies, giving a strong basis to the study.^40^ Previous studies have assessed the effectiveness of similar interventions on asthmatic patients’ adherence and found a similar increase between 10% and 40%.^41,42^ However, this increase mainly occurred up to 10 weeks after follow-up but decreased after 26 weeks. The 51.8% increase in Study 2 is larger than previous studies, which could be attributed to the core components of the intervention, continuous follow-up by the professionals and monitoring of the enrolled participants. In future studies, it is worth noting that a high level of monitoring and follow up is needed, and chronic disease management being delivered on a regular basis rather than a one-off intervention, could help build rapport and trust with the participants.

Improvements in SBP were not statistically significant by the end of Study 2. This could be attributed to the low baseline BP levels of patients in the study and not including those with uncontrolled hypertension. It is also crucial to consider the length of time it takes for BP changes to present, especially since changes in DBP only started showing 5 months after follow-up. However, this study was able to produce statistically significant improvements in the mean CCQ scores from the third visit until the end of the study follow-up period of 12 months. One of the study’s major strengths likely contributed to this improvement: providing a tailored, patient-centred intervention based on evidence-based frameworks that acknowledge no single theory is effective in all patients. Nevertheless, findings of this study are insufficient to make a firm conclusion on what pharmacist interventions are effective in patients with co-existing COPD and hypertension; this is a significant gap in literature found in this systematic review.

Study 3 was associated with statistically significant improvements in both patient and health economic outcomes.^21^ Previous meta-analyses have already concluded a pharmacist-led intervention can produce changes in SBP, consistent with this study’s reduction of SBP.^43-45^ However, like Study 2, Matzke et al., included patients diagnosed with any stage of hypertension, rather than exclusively uncontrolled hypertension patients.^22^ Although this was a limitation in Study 2 that may have not led to significant changes in SBP, it is considered a strength in Study 3 as it creates a potential program for all hypertensive patients. It is important to note however, that participants recruited in the collaborative care group may have been less healthy than the usual care patients, especially since they were given the opportunity to participate while being hospitalised or after referral to a medical professional.

The main limitation of Study 3 llies in its limited reporting of clinical outcomes, focusing on analysing secondary economic outcomes including ED utilisation among the collaborative care patients. Although an inclusion criterion of this study was patients diagnosed with two or more conditions including hypertension and COPD, the study does not report any changes seen in COPD. This raises concern whether the intervention in this study is strictly effective for hypertension only, rather than both co-existing conditions. During this one-year study, there were also several changes made to the hypertension management guidelines. For instance, the SBP goals in patients above the age of 60 was increased from 130 to 150mmHg; this probably confounded with the effect of the intervention, especially since the change may have reduced the clinicians’ assertiveness in lowering the patients’ BP and making the cut-off. This is particularly concerning since the study design was not a randomised controlled trial, but rather a retrospective analysis and assignment of control group participants may have included results based on previous hypertension guidelines, raising a risk of inaccuracy of findings.

A strength of this review is the comprehensive search of major databases and trial registries for any future or in-progress studies, as well as reference tracking. However, despite this thorough search, only three studies were found that included the target population, people with concomitant COPD and hypertension. This review condenses the literature available not only on COPD and hypertension outcomes but also examines economic impact, summarising the results achieved and analyses the gaps in the data found.

Despite the strengths, it is important to acknowledge the risk of bias in the studies included for this review, one of which was considered high risk. This reinforces the scarcity of the literature available on managing concomitant COPD and hypertension, making this review and similar studies in the future crucial to addressing the issue. Another limitation of the studies included is that although a diagnosis of COPD and hypertension was in the inclusion criteria of all studies, Study 3 only reported changes in BP and failed to include any COPD-related outcomes, which raises questions as to whether the intervention was in fact effective in improving both conditions rather than one. Furthermore, citation tracking and search of grey literature might have enhanced the yield of relevant records in this review.

Future studies should focus on filling the research gap unearthed by this review, by developing pharmacological and non-pharmacological interventions targeted at both COPD and hypertension. The dearth of relevant studies included in this review highlight the need for high-quality, randomised controlled clinical trials that aim to improve health outcomes of people with both conditions. Recent publications have been addressing the burden of major adverse cardiovascular events (MACE) in COPD, which is a positive first step towards addressing the issue and subsequently finding solutions.^46,47^ Future research should also focus on current treatment regimens recommended for each condition individually and assess their effectiveness and safety when these conditions co-exist. The ongoing Phase III THARROS trial is comparing the effectiveness of triple ICS/LAMA/LABA therapy compared to LAMA/LABA therapy in participants with COPD and elevated cardiopulmonary risk.^48^

In conclusion, this systematic review found limited evidence on interventions for managing concomitant COPD and hypertension. The three included studies found contradictory evidence on the effectiveness of interventions on reducing SBP, but two of the studies established an improvement in COPD 6MWD in the intervention group. Despite practitioners encountering patients with both conditions on a regular basis, research remains scarce in developing a treatment algorithm that includes a stepwise approach to manage both conditions concurrently. Best practice currently would be to manage both conditions according to relevant guidelines. Although there is growing literature promising to bridge this gap, there remains an urgent need for research studies that test interventions to improve health outcomes for the millions of people living with COPD and hypertension.

## Supplemental Material

Supplemental Material - Interventions for the management of concomitant COPD and hypertension: A systematic review Supplemental Material for Interventions for the management of concomitant COPD and hypertension: A systematic review by Sadan Taher, Aletta E. Schutte, John R. Hurst, Chris P. Gale and Sameera Ansari in Journal of Multimorbidity and Comorbidity

## Data Availability

Data sharing is not applicable to this article as no datasets were generated or analysed during the current study as a systematic review. *
