# Supplementary material for: Interventions for the management of concomitant COPD and hypertension: A systematic review
Source: J Multimorb Comorb. 2025 Jun 23;15:26335565251341389. doi: 10.1177/26335565251341389 (PMC12185945; doi:10.1177/26335565251341389)
Supplement: Supplemental Material - Interventions for the management of concomitant COPD and hypertension: A systematic review [file sj-pdf-1-cob-10.1177_26335565251341389.pdf]

Supplement 1: Search Strategy

No.	Database	Group	Search query	Results
1	**Scopus**	Syntax	(copd OR "chronic obstructive pulmonary disease") AND hypertension AND intervention AND implementation AND (LIMIT-TO (LANGUAGE , "English"))	3,148
2	**CINAHL**	Syntax	TX (copd or chronic obstructive pulmonary disease) AND TX hypertension AND TX intervention AND TX implementation	45
		Limits	English Language	
3	**PubMed**	Syntax	(copd OR "chronic obstructive pulmonary disease") AND hypertension AND intervention AND implementation	64
		Filters	English	
4	**Embase**	1	(copd or "chronic obstructive pulmonary disease")	31
		2	exp hypertension/ or exp elevated blood pressure/ or exp essential hypertension/	
		3	1 and 2	
		4	intervention	
		5	3 and 4	
		6	implementation	
		7	5 and 6	
		8	limits 7 to English language	
				100
5	**Cochrane Library**	1	COPD OR "chronic obstructive pulmonary disease"	
		2	Hypertension	
		3	Intervention	
		4	Implementation	
		5	1 and 2 and 3 and 4	

**6**	**Cochrane Central Register of Controlled Trials**	1	COPD OR "chronic obstructive pulmonary disease"	30
		2	Hypertension	
		3	Intervention	
		4	implementation	
		5	1 and 2 and 3 and 4	
			**Total number of records identified from search of the above databases**	**3,418**

Supplement 2: Study Risk of Bias Assessment

Study ID	D1	D2	D3	D4	D5	Overall
1.Alasmari et al., 2024						
2.Torres-Robles et al., 2021						
3.Matzke et al., 2018						

Key

	Low risk
	Some concerns
	High risk

D1	Randomisation process
D2	Deviation from the intended intervention
D3	Missing outcome data
D4	Measurement of the outcome
D5	Selection of the reported result
